# Spousal violence against women and its consequences on pregnancy outcomes and reproductive health of women in India

**DOI:** 10.1186/s12905-021-01515-x

**Published:** 2021-11-01

**Authors:** Mahadev Bramhankar, R. S. Reshmi

**Affiliations:** Department of Migration and Urban Studies, International Institute for Population Sciences (IIPS), Mumbai, 400088 India

**Keywords:** Adverse pregnancy outcome, Reproductive health, Spousal violence, Sexual violence, Physical violence

## Abstract

**Background:**

Globally, one in three women experienced domestic violence. Alike the scenario observed in India, and a very few studies talk about violence and its consequences on women's health. Hence, the purpose of this study is to access the level of various types of spousal violence in India and to understand the association between physical, sexual and emotional violence against ever-married women by their husbands. The study further examines the consequences of spousal violence on women's health in terms of adverse pregnancy outcomes and reproductive health in India.

**Methods:**

The study uses secondary data from National Family Health Survey-4 (NFHS-4, 2015–16). The analysis was based on a sample of ever-married women aged 15–49 years. Bivariate descriptive analysis and multiple regression analyses have been carried out to understand the association between spousal violence and its consequences on women's health.

**Results:**

The study finds that the physical, sexual and emotional violence experienced by ever-married women in India are 29.8%, 13.8% and 7.0%, respectively. Further, the physical and sexual violence experienced by women have a significant association with an unwanted pregnancy, abortion, miscarriages and ever had termination of pregnancies. The regression analysis shows that violence by sexual partners among battered women increased the likelihood of unwanted pregnancy. Similarly, abortion and ever had a termination of pregnancies are also adversely affected by partner violence. Further, the risk of sexually transmitted infection increases 77% by sexual violence and 44% by emotional violence among battered women. Also, Sexual violence substantially increases the risk of prolonged labour during pregnancy.

**Conclusion:**

This study revealed that one in three women experiencing violence by their husband and also it is evident that various forms of spousal violence adversely affect pregnancies outcomes and reproductive health among battered women compared to not battered.

## Introduction

A multi-country study done by the World Health Organization (WHO) revealed that physical violence ranged from 12.6% in Japan to 61% in Peru. Ethiopia and Peru have a substantial prevalence of sexual violence, ranging from 48 to 59%, whereas Japan has the lowest pervasiveness [1]. In India, 33% of the ever-married women aged 15–49 experienced physical violence, 7% had experienced sexual violence, and 13% had the experience of emotional violence by their current or former husband throughout married life [2]. According to the existing literature, one of the adverse effects of spousal violence could be unintended pregnancy, as those women might suffer from sexual violence and be afraid to ask or discuss contraception with their husbands [3]. The same study also stated that prevalence of unintended pregnancy is higher among physically battered women by a partner. Women experiencing physical violence by the husband were more likely to report the risk of unwanted pregnancies resulting in a live birth [4]. Another study of pregnant women of the postpartum period in Peru found that 65% of the pregnancies were unintended. Those who experienced any life abuse (physical or sexual) had a higher risk of unintended pregnancies [5].

According to an estimates of the WHO, maternal deaths and 4.6 million disability-adjusted life year (DALYs) could prevent worldwide causes if they avert unintended pregnancies [6]. Another study showed a significant positive relation between spousal violence and unintended pregnancy [7]. Further, in the context of patriarchal municipalities found that women living in municipalities with higher intimate spousal violence levels had the risk of almost three times the odds of having unintended pregnancies [8]. Nearly one fourth of the married women reported having experienced an unintended pregnancy in the last five years of the survey. The same study also reported that among ever battered women by their husbands' sexual form of violence were 2.3 times higher chances of unintended pregnancy than women who did not experience sexual violence [9]. Perhaps another study showed that miscarriages were more exposure in the physically battered women than sexually battered women as they would fall, beaten or threatened at the time of pregnancy. A study done by Kishor and Jonhson [10], reported that spousal violence positively associated with the probability of poor reproductive outcomes when wealth and other factors are held constant. Among the ever pregnant women, 23% experienced involuntary pregnancy loss, while 7% reported induced abortion among those who had exposure to intimate spousal violence [11]. Intimate Partner Violence (IPV) affected woman's physical and reproductive health, reduced sexual autonomy, and increased risk of unintended pregnancy and abortions [12].

Similarly, the study by Silverman [13, 14], reveals that adjusted regressions results significant associations between miscarriage and IPV Adjusted Odds Ratio (AOR = 1.35, 95% CI = (1.11,1.65) and stillbirth (AOR = 1.36, 95% CI = (1.02,1.82) ever, as well as with labour complications with AOR 1.27 [13, 14]. Women who reported physical and sexual violence were 1.7 times more likely to report gynecologic morbidity [15]. For the several clinical based studies conducted in various part of the globe especially developing regions indicates a strong association of intimate spousal violence and symptoms of Sexually Transmitted Infection (STI) with reproductive health problems [16]. Another study in Vietnam that shore up confirms the relationship between experiencing violence and being at high risk of STI and HIV. The estimated proportions of intimate partner violence (IPV) during pregnancy ranged from 5.9% to 32.5%. Depending on the form of IPV, it is strongly associated with a greater risk of both mental disorders and adverse birth outcomes (low-birth-weight and preterm labour) [17]. Ever married women who experienced violence have self-reported poor reproductive health compared with not battered women [16]. In Bangladesh, women who experienced sexual IPV during pregnancy were at increased risk of suffering from IPV during pregnancy are positively associated with pregnancy complications [18].

There are many studies that reported the relation between women who experienced violence and adverse pregnancy outcomes and reproductive health faced by married women. Even though very few studies address this problem in the Indian context. The nationally representative NFHS-4 data has opened the window of opportunity for scientific investigation of battered women through various socio-demographic, economic and other characteristics. Therefore, the present study focuses on the attributable effect of spousal violence on ever-married women's pregnancy outcomes and reproductive health. In the present study, firstly we estimate the prevalence of various types of spousal violence across the different socio-demographic characteristics and also states wise differential for the ever-married women aged 15–49 years in India. Moreover, the secondary objective of this study is to explore the association of physical, sexual and emotional spousal violence on adverse pregnancy outcomes and reproductive health of Indian women.

## Material and methods

This study is based on a large scale cross-sectional survey well known nationally representative data of the National Family Health Survey fourth round (NFHS-4). The NFHS is also referred to as the Demographic Health Survey (DHS) in other countries' perspectives. It is conducted regularly in many developing countries to obtain population-based estimates of major health issues, family planning and violence-related information collected from various countries. Data was collected for NFHS-4 in all 29-states and seven union territories during the 2015–2016 period. In India, NFHS-4 gives information at the district level, but the survey included a section on 'Domestic Violence', representing the state level. The information was collected from only one woman per each household. The present study restricted the sample of ever-married women aged 15–49 years, including currently married, divorced, separated, widowed, and no longer living together. The sample size related to spousal after giving weight, the ultimate sample related to spousal violence was 61,906 ever-married women. The term 'domestic violence' module of the NFHS-4, spousal violence, uses a modified version of the Conflict Tactics Scales (CTS) [19, 20]. The same study includes questions that have asked women whether their current or most recent (if divorced, separated, or widowed) husband/partner ever perpetrated any series of behaviorally specific acts of physical, emotional, or sexual violence. Those who reported that they ever experienced any violence have been categorized as any experienced of spousal violence by husband or partner.

### Definitions of key measures used in the analysis

The present study uses various types of spousal violence, such as physical violence, emotional violence, sexual violence, and any violence which are defined here, according to the given Demographic Health Programmed criteria [21]. For the computation of physical violence, a set of seven-question were asked to ever-married women. If a woman reported that they experienced any of the following experienced by women by husband or partner considered as a case of physically abused women, and the questions were such that: (a) Slap you?, (b) Twist your arm or pull your hair?, (c) Push you, shake you, or throw something at you?, (d) Punch you with his fist or with something that could hurt you? (e) Kick you, drag you or beat you up? (f) Try to choke you or burn you on purpose? (g) Threaten or attack you with a knife, gun, or any other weapon?. Similarly, for the computation of Emotional violence, asked three questions to the ever-married women have asked: (a) Say or do something to humiliate you in front of others? (b) Threaten to hurt or harm you or someone close to you? (c) Insult you or make you feel bad about yourself? If one of the answers positively responds to women considered as a case of emotional violence. Further, for the computation of sexual violence, three-question responses were asked, (a) Ever been physically forced into unwanted sex by husband/partner? (b) Ever been forced into other unwanted sexual acts by husband/partner? Ever been physically forced to perform sexual acts respondent didn't want?. Based on the aforementioned different types of violence, another term called any spousal violence is a combination of any kind of physical or emotional or sexual violence experienced by women in their married life.

In our study, the information about abortion, and miscarriages have collected from those women who had a history of pregnancy in the last five year. For the prolonged labour problem, the question "During delivery, did you experience prolonged labour?" was asked to the respondent who has history of pregnancies in last five years. Further, the information about ever termination of pregnancy was asked to all married women in the age group 15–49. This study also signifies the problem of reproductive health which was considered as a Sexually Transmitted Infection (STI) in the last 12-months reported by all ever-married women. Furthermore, in the study, additional explanatory factors considered as derived from prior literature included as the residence, age group, religion, socio-economic status, caste, intergenerational violence, children ever born, contraception use and their education [11, 22, 23].

The present study examine the association among various factors, descriptive statistics and possible associations between factors were explored by conducting cross-tabulations. Further, the multivariate logistic regressions were used to obtain adjusted odds ratios (AORs) by controlling for the more commonly recognized explanatory factors using household characteristics such as residence, wealth index, religion and caste. The P-value below 0.05 was considered statistically significant, at 95% of the confidence interval. An odds ratio larger than one represents a greater likelihood of the outcome than for the reference category and vice-versa for multiple logistic regression results. Using STATA version.15 and Excel software were used for data analysis.

## Results

**Fig. 1:**
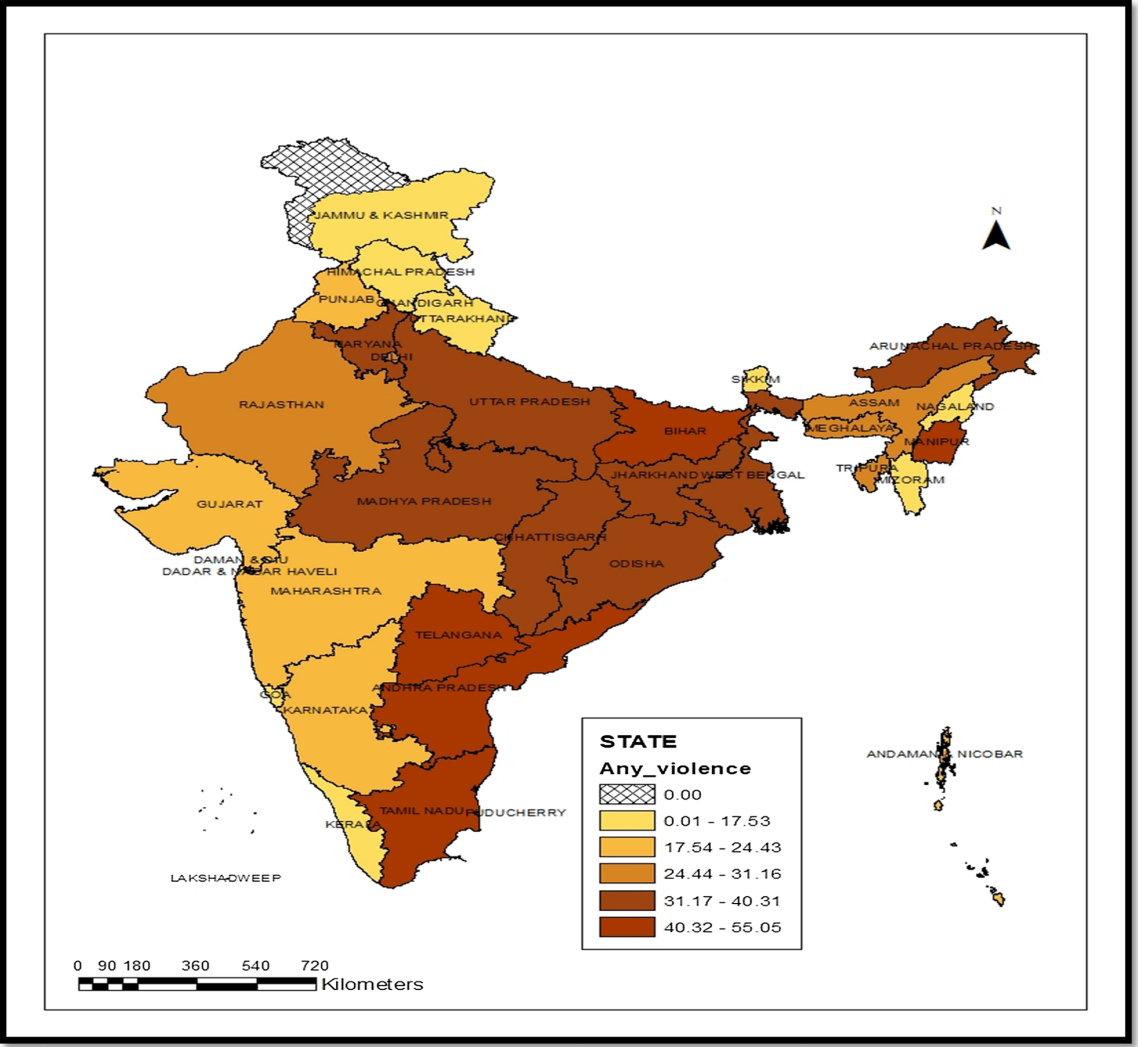
Any type of violence across states of India (%), NFHS-4 (2015–16). *Note*: The data for Jammu & Kashmir area was not available which showed by cross line in this figure

Table 1(a) and (b) gives the prevalence of various form of spousal violence by their household and socio-economic and demographic characteristics. It is evident from the table that, in India, 29.8% of the women reported physical violence, 13.8% reported emotional violence and 7% reported sexual violence and the prevalence of any of these violence was 33.3%. Further, as shown in Fig. 1, the prevalence of spousal violence ranged from 3.5 to 55% across the states and union territory, showing ta significant difference in the level of spousal violence across the country. Manipur has the highest, and Sikkim has the lowest prevalent of spousal violence in India. The household characteristics such as type of place of residence, wealth, caste, and religion were significantly associated with the various forms of violence, on the same line, intimate spousal violence related to women's individual-level characteristics in India.

**Table 1:** Prevalence of various type of spousal violence among ever-married women and its association with (a) household characteristics, (b) women characteristics

	Physical violence	Emotional violence	Sexual violence	Any violence	n
*(a) Household characteristics*					
Place of residence	***	***	***	***	
Urban	24.3	12.0	5.2	27.7	21,461
Rural	32.7	14.8	7.9	36.3	40,445
Wealth Index	***	***	***	***	
Poorest	42.2	19.2	11.6	46.0	10,550
Poorer	36.8	16.5	8.4	40.7	11,974
Middle	31.4	15.0	7.1	35.3	12,793
Richer	25.7	12.0	5.3	29.1	13,140
Richest	16.2	8.0	3.5	19.1	13,449
Religion	***	***	***	***	
Hindu	30.5	14.0	7.0	34.0	50,215
Muslim	27.1	14.1	6.7	31.5	8449
Christians	28.8	14.0	7.2	33.3	1474
Others	21.8	7.9	6.5	23.3	1769
Caste	***	***	***	***	
Schedule caste (SC)	37.1	17.4	9.1	40.6	12,050
Schedule tribe (ST)	32.7	15.6	8.7	36.5	5684
OBC	30.8	14.1	6.8	34.6	27,350
Others	21.9	10.3	5.1	25.0	16,823
India	29.8	13.8	6.96	33.3	61,906
*(b) Women's characteristics*					
Age Interval	***	***	***	***	
15–24	24.19	11.98	6.41	11.98	11,356
25–34	30.32	13.55	7.24	13.55	22,967
35–44	31.59	14.59	6.98	14.59	19,164
45–49	31.70	15.41	6.91	15.41	8418
Children ever born	***	***	***	***	
0	18.6	11.6	5.7	23.0	6123
1	23.1	12.1	6.0	26.3	11,407
2	28.8	12.8	5.9	32.1	20,198
3+	36.6	16.1	8.7	40.3	24,178
Women's education	***	***	***	***	
No education	39.7	18.4	9.4	43.6	20,114
Primary	36.5	15.8	8.5	39.7	8847
Secondary	24.1	11.5	5.6	27.8	26,522
Higher	12.8	6.3	2.9	15.0	6423
Current marital status	***	***	***	***	
Married	29.2	13.2	6.7	32.8	58,480
Widowed	29.9	14.3	8.5	31.9	2477
Divorced	60.9	54.2	24.8	65.8	289
No longer living together	68.5	54.5	21.1	75.4	661
Intergenerational Violence	***	***	***	***	
No	23.2	10.6	5.1	26.3	46,780
Yes	53.1	25.4	13.5	57.7	13,011
Don’t know	33.1	13.8	7.5	38.2	2115
Contraception use	***	**	*	***	
Not using	28.0	14.0	7.3	31.9	28,507
Using	31.3	13.7	6.7	34.5	33,399

Significant level: ***p ≤ 0.001; **p ≤ 0.01;*p ≤ 0.05

n is un-weighted sample

Table 2, indicates the association between various types of spousal violence and health problem experienced by women in India. It revealed that the proportion of women who reported abortion and miscarriages are 4% and 5.7% respectively. Similarly, nearly one fifth of the women reported that they ever had terminated pregnancy. Among the currently pregnant women, 12.3% has reported to have unwanted pregnancy. The prevalence of STI among women was 2.43% in the last 12 months of the survey. Furthermore, 42.3% of women experienced prolonged labour during pregnancy. Table 2 shows that the currently unwanted pregnancy, abortion and ever had a termination of pregnancies have a strong association with physical, emotional, sexual violence.

**Table 2:** Percentage of adverse pregnancy outcome, sexual and reproductive health among ever-married women in age group 15–49 and its association with various forms of spousal violence

Exposure of violence	STI (in last 12 months)		Current unwanted pregnancy		Abortion		Miscarriage		Ever had a termination of pregnancy		Prolonged labour during pregnancy	
Spousal violence	(%)	n	(%)	n	(%)	n	(%)	n	(%)	n	(%)	n
Physical	***		***		***		*		***		***	
No	2.25	43,472	11.05	2698	3.0	16,026	5.65	16,026	14.53	43,472	40.58	15,127
Yes	2.83	18,435	16.18	836	5.0	6755	5.91	6755	20.34	18,435	47.49	6306
Emotional	***		***		***		**		***		***	
No	2.24	53,341	11.68	3114	3.3	19,737	5.49	19,737	15.45	53,341	41.53	18,630
Yes	3.59	8565	16.61	420	5.7	3044	7.26	3044	21.33	8565	49.85	2803
Sexual	***		***		***		*		***		***	
No	2.28	57,597	11.54	3302	3.4	21,091	5.82	21,091	15.79	57,597	42.03	19,873
Yes	4.37	4309	22.52	231	6.3	1690	4.61	1690	22.63	4309	50.09	1560
Any violence	***		***		***		**		***		***	
No	2.17	41,278	10.62	2542	2.9	15,236	5.55	15,236	14.31	41,278	40.18	14,404
Yes	2.95	20,628	16.46	992	4.9	7545	6.08	7545	20.17	20,628	47.60	7029
India	2.43	61,906	12.26	3534	4	22,781	5.73	22,781	16.26	61,906	42.61	21,433

Significant level ***p < 0.001; **p < 0.01; *p < 0.05

n is un-weighted sample

The multivariate logistic regression model was adjusted by household characteristics given in the Table 3. The result shows, (AOR 1.72, 95% CI 1.19, 2.5), that the likelihood for the unwanted pregnancy was higher among women who experienced any sexual violence. Unwanted pregnancy was comparatively higher among women who experienced sexual violence as compared to those who faced physical and emotional violence. The analysis reveals that the nearly 61 percent of the physically battered and 53 percent of the sexually abused women had experienced abortion. Similarly, the likelihood of experiencing termination of pregnancy was higher among women who experienced physical and sexual violence. The problems such as sexually transmitted diseases (STI) in the last 12 months and prolonged labour during pregnancy were positively associated with spousal violence, which affects the sexual and reproductive health of women. The risk of prolonged labour during pregnancy was significantly higher among women who experienced physical, sexual, and emotional violence. The multivariate adjusted model, shows that the risk of STIs is 77% higher among women who experienced any sexual violence (AOR = 1.77, 95% CI 1.47, 2.1) and 44% higher among women who experienced any emotional violence (AOR = 1.44, 95% CI 1.23, 2.67).

**Table3:** The association for the health consequences on women and exposure of physical, sexual, and emotional violence in India

Exposure of violence	STI (in last 12 months)			Unwanted pregnancy			Abortion			Ever had a termination of pregnancy		
	AOR	95% C.I		AOR	95% C.I		AOR	95% C.I		AOR	95% C.I	
*Physical*												
No	1			1			1			1		
Yes	1.11	0.97	1.27	1.22	0.94	1.57	1.61***	1.36	1.91	1.43***	1.35	1.50
*Emotional*												
No	1			1			1			1		
Yes	1.44***	1.23	1.67	1.01	0.72	1.41	1.36**	1.10	1.66	1.18***	1.10	1.25
*Sexual*												
No	1			1			1			1		
Yes	1.77***	1.47	2.10	1.72**	1.19	2.50	1.53***	1.20	1.93	1.24***	1.14	1.34
*Place of residence*												
Urban	1			1			1			1		
Rural	1.06	0.94	1.20	1.22	0.92	1.61	0.87	0.74	1.03	0.87**	0.82	0.91
*Wealth index*												
Poorest	1			1			1			1		
Poorer	1.45***	1.19	1.78	0.84	0.63	1.12	1.52**	1.19	1.95	1.12**	1.05	1.21
Middle	1.83***	1.51	2.23	0.80	0.60	1.08	1.99**	1.56	2.54	1.08	1.00	1.16
Richer	2.09***	1.70	2.55	0.56**	0.39	0.79	2.27**	1.76	2.92	1.13**	1.05	1.22
Richest	2.67***	2.16	3.30	0.43***	0.28	0.64	2.11**	1.58	2.79	1.16**	1.06	1.26
*Religion*												
Hindu	1			1			1			1		
Muslim	0.99	0.84	1.15	1.72***	1.32	2.21	0.89	0.73	1.09	1.21***	1.13	1.28
Christians	0.63	0.43	0.94	0.42	0.14	1.24	0.70	0.42	1.19	0.90	0.78	1.04
Others	0.87	0.63	1.18	1.09	0.51	2.34	0.69	0.41	1.16	0.86*	0.74	0.98
*Caste*												
OBC	1			1			1			1		
SC/ST	1.14	1.00	1.30	1.01	0.79	1.30	0.89	0.75	1.07	0.95*	0.89	1.00
Others and don’t know	1.21	1.07	1.38	1.06	0.81	1.39	1.19	0.98	1.46	1.07*	1.01	1.13

Ref: First category as the Ref Category with AOR 1: (*AOR* Adjusted Odds Ratio), C.I. 95% confidence interval

P-value with level of significance: ***p ≤ 0.001; **p ≤ 0.01;*p ≤ 0.05

## Discussion

The present study explores the level of spousal violence experienced by the women in India and its consequences on the sexually transmitted diseases, prolonged labour, unwanted pregnancy, abortion, ever had a termination of pregnancy and miscarriages [24, 25]. This study reveals that one among three women experienced any kind of violence in India by her partner or husband, which is on the same line with other studies [1, 23, 26]. The study further focuses on any type of spousal violence given by various socio-demographic, economic and other covariates backgrounds. All factors were significantly associated with physical, sexual and emotional spousal violence [27, 28]. Moreover, this study has also extended the prevalence based analysis at the state level for any spousal violence. The state-level spousal violence revealed that northeast region states (Manipur, and Aruanachal Pradesh), eastern states (Bihar, Uttar Pradesh and other) and southern (Telangana, Andhra Pradesh, Tamil Nadu) shows higher prevalence compared to the national level and other states of India.

The violence against women could significantly affect women's physical, mental, and sexual health. The present study indicates that women who experienced any physical, sexual and emotional violence are more likely to have abortion, which is on the same line with other studies conducted in different regions [13, 14, 29]; the risk of unintended pregnancies is 1.72 times higher among women who experienced any sexual spousal violence, and this results is mentioned in previous studies [5, 30, 31]. Women who faced sexual and emotional violence are at higher risk of STI Infections which are in line with similar studies [12, 32, 33]. When we discuss about the risk of prolonged labour during pregnancy delivery observed higher among battered women in India [34]. This study also demonstrates the higher prevalence of unwanted pregnancy and ever had a pregnancy termination among women those who experienced physical or sexual violence in their married life by husband [11, 35].

Cumulatively, as per the present study in the Indian context and existing studies in various subcontinents, it shown that there is an attributable impact of spousal violence on the health of ever-married women, specifically in reproductive and pregnancy [36]. These findings were based on cross-sectional data and opened the scope for further strong cohort-based experimental study. In India, the level of domestic and spousal violence has been a decline but still it at a higher level where one in three women are battered by her husband. It is still an issue of concern as its adverse consequences not only on the physical health of women but also mental and social health. Ongoing and existing studies were revealed that spousal violence is mainly experienced by a wife by her husband. A country like India, where the more prevalent nature of patriarchal society intends to women usually has a secondary place after her husband. In Indian households, mostly in rural areas, it has been seen that men have been dominant women regarding decision making, facing injustice, oppression, suppression, and exploitation in male-dominated Indian society [37, 38]. Therefore, in the subcontinent like, India should have multi-sectoral approaches in some kind of women empowerment, domestic violence law reforms and women security against spousal violence. In married women, reducing the level of such adverse reproductive and pregnancy problems can be reduced by the decline in the level of spousal violence against women in India.

## Conclusions

This study provides evidence-based on population level survey data of abortion, currently unwanted pregnancies, sexual and reproductive health problems among married women and its association with IPV among women in India. The present study has included socio-economic and demographic indicators and other covariates such as children ever born, contraception use and intergeneration exposure to violence among women during their childhood, shown strong predictors of spousal violence. The analysis indicates that spousal violence exposure among ever-married women affects women's health, particularly the reproductive health and pregnancy outcomes which affected by physical, sexual or emotional abused. This study clearly indicates that sexual and physical violence contributed to the significant factor for pregnancies loss and unwanted pregnancies. Finally, to conclude this study, we can say that there is a significant difference in the health consequences or adverse pregnancy outcomes among battered and not battered women. Any form of spousal violence such as physical, sexual and emotional violence are increases the public health burden. The above results advocate that preventing physical, emotional and sexual IPV can improve maternal health and pregnancy outcomes among the ever-married women in India. In India, for preventing this, at the national and sub-national level should have multi-sectoral approaches such as women empowerment and higher education, strict law against domestic violence and women security.

### Limitation of the study

This study has quite a few limitations that have to be acknowledged. This study is based on cross-sectional data and does not allow the sequencing of events to demonstrate a causal association. The data for the domestic violence of married women is too sensitive. It again depends on women's perception whether she provides information indeed or not about their sensitive private matter. A slap is unacceptable to some women, while for some other women, it may be acceptable, and hence, it would be under-reported. Spousal violence depends on many different social and community-level factors such as crime rates in society, government strictness, response to Women's crime reported by police and various things related to spousal violence, which not consider in NFHS-4 survey data. Apart from that, the question related to sexually transmitted infection is self-reported and not tested clinically. If available just after experiencing violence by women, the information about STI to women only for the last 12 months may give good results.

## Data Availability

The study's datasets are publicly available Demographic Health Survey (DHS) site.

## Abbreviations

IPV: Intimate Partner Violence
NFHS: National Family Health Survey
AOR: Adjusted odds ratio
STI: Sexually transmitted infection
